# Comparative study on slaughter performance, meat quality, and rumen microbiota of Hainan Dong goat and its hybrid with Nubian goat

**DOI:** 10.3389/fvets.2024.1447321

**Published:** 2024-10-22

**Authors:** Huiyu Shi, Naifeng Zhang, Yan Tu, Yanhong Yun, Qiyu Diao, Tao Ma

**Affiliations:** ^1^Key Laboratory of Feed Biotechnology of the Ministry of Agriculture and Rural Affairs, Institute of Feed Research of Chinese Academy of Agricultural Sciences, Beijing, China; ^2^Department of Animal Science, School of Tropical Agriculture and Forestry, Hainan University, Haikou, China

**Keywords:** goat, hybrid, performance, meat quality, rumen microbiota

## Abstract

**Introduction:**

The Hainan Dong goat (DG) is a local meat breed widely raised in Hainan, China because of its good adaptability to local hot and humid weather. However, the growth rate of these DG is much slower than that of commercialised breeds improved in European countries, resulting in poor carcase characteristics, including smaller slaughter weight and carcase weight, which have become increasingly prominent. In recent decades, Nubian goats have been continuously imported into China to improve the production performance of local breeds.

**Methods:**

In this study, the effects of breed on growth performance, carcase and meat quality, and ruminal microbiota were analysed in 40 goats, including 20 DGs and 20 hybrid F3 offspring generated by crossing the DG and Nubian hybrids (NH). All the goats were averagely aged 90 days and weighed at 11  ±  1.34  kg. They were assigned to two treatments, with three replicates per treatment. The two groups were fed the same diet for 90 days before slaughter.

**Results:**

The results showed that the average daily gain, F/G ratio, slaughter weight, and carcase weight of the NH group were higher than those of the DG group (*p* < 0.05). However, tube circumference, meat-to-bone ratio, and eye muscle pH were lower in the NH group than in the DG group (*p* < 0.05). The NH group showed a smaller fibre crosssectional area and fibre diameter, but a larger fibre density than the DG group (*p* < 0.05). Bacteroidetes and Firmicutes were the most dominant phyla in the two groups; however, the two breeds had different ruminal microbial communities.

**Discussion:**

In the present study, the differences in growth performance between two groups of goats under the same feeding environment and feed conditions were compared. The correlation between feed sources and rumen flora has been demonstrated, and the results of this study show that the same diet has similar effects on rumen microorganisms, which in turn have related effects on growth and production performance.

**Conclusion:**

In summary, hybrids can improve the growth and slaughter performance of local breeds, which may be related to changes in the rumen microorganisms. This study revealed that crossbreeding of Nubian goats with Dong goats has the potential to be used in a wide range of applications owing to its effectiveness in increasing production efficiency.

## 1 Introduction

Goats are one of the most adaptive and important livestock species, and are good sources of lean meat. From a nutritional perspective, the proportion of polyunsaturated fatty acids in goat tissues is higher than that in sheep tissues (1). The Hainan Dong goat, known as the Dong goat, is a local meat breed widely raised in Hainan, China because of its good adaptability to local hot and humid weather (2). DG meat is also popular in southern China because of its delicious flavour. Previously, the DG grazed freely. Recently, goats have been restricted to protect the environment and develop large-scale farming. Although this breed of goat has great flaws, such as slow growth rates and small body sizes, goat meat remains very popular in southern China because of its delicious flavour. It is not only characterised by tolerance to crude DG feed but also by a higher resistance to parasitic diseases and thinner muscle fibres (3). However, the growth rate of these DG is much slower than that of commercialised breeds (4) improved in European countries, resulting in poor carcase characteristics, including smaller slaughter weight and carcase weight (5), which have become increasingly prominent. The demand for goat production will continue to increase in the future as people pursue healthy living standards that will seriously restrict the rapid development of the Hainan goat industry.

Although the demand for DG exceeds its supply in production, the economic benefit of the pure DG breed is low because of its small size, relatively slow growth rate, long-term lack of scientific breeding, and serious breed degradation. To improve the economic benefits of Hainan goat-breeding enterprises and increase the supply of mutton to the Hainan market, Nubian black goats were hybridised with DG. Nubian goats are native to Africa and widely distributed in the arid and extremely arid areas of northern Sudan (6). They are considered world-famous dual-use meat and milk breeds, but are susceptible to parasites. Owing to its unique advantages of large size and fast growth, it is particularly suitable for crossbreeding and improving local varieties (7). In recent decades, Nubian goats have been continuously imported into China to improve the production performance of local breeds (8). In a study by Meza-Herrera et al., local goats of a non-defined breed were recurrently crossed with imported sires of Granadina, Nubian, Alpine, Saanen, and Toggenburg breeds. The results showed that the largest litter size at birth was for animals upgraded to Nubian, followed by Granadina. The heterosis obtained from the Nubian cross, the only significant cross, was much larger (9). Zhang et al. identified candidate genes and miRNAs associated with muscle development and indicated their potential roles in muscle fibre hypertrophy. These findings will bolster further studies on goat muscle development and molecular breeding, which may explain the continuous import of Nubian goats into China with high meat yields over the past decades to improve the meat yield of other goat breeds (10).

Based on previous studies and rarity of DG (11), it was hypothesised that hybridisation might improve the body shape and external characteristics of local DG and enhance their growth performance and carcase characteristics. Therefore, this study aimed to assess the effects of hybridisation on production performance, slaughter performance, meat quality, and rumen microorganisms in a local DG. This study compared the changes in the above indices of Hainan native DG and hybrid F3 offspring generated by crossing the NH, providing a scientific basis for breeding DG and crossbreeding.

## 2 Materials and methods

This study was performed by following the protocols approved by the Animal Ethics Committee of the Institute of Feed Research of the Chinese Academy of Agricultural Sciences (permission number: IFR-CAAS20220808) in Beijing, China, and all experimental plans and procedures were supported. Academic standards for animal use in research were strictly followed. A completely randomised experimental design was used in this study.

### 2.1 Animals and experimental design

Forty castrated male goats born in the same season, including 20 DG kids and 20 crossbred with Nubian black goats, were selected, with an initial age of 90 ± 10 days and an initial body weight of 11.45 ± 1.2 kg. All goat kids were weaned at 3 months and began to enter the fattening stage. The treatments included a control group (DG) and an experimental group (NH, hybrid F3 offspring), with three replicates per treatment; each replicate was 7, 7, and 6. The two groups were fed the same diet for 90 d before slaughter.

Pennisetum hydridum and peanut straw were used as roughage (1:1), and the composition of the concentrate is shown in Table 1. Feed was prepared according to the feeding standards for meat-producing sheep and goats (NY/T816-2004). The goats were kept in separate corrals, and the experimental sheds were well-ventilated and clean. The goats were fed twice (at 07:00 and 18:00) and had free access to water. The concentrate-to-forage ratio of the basal diet was 7:3. The transition period lasted 7 days, and the trial period lasted 90 days.

**Table 1:** Ingredients and chemical composition of the basal diet (%, as-fed basis).

Ingredients	Content	Nutrient levels	Content
Pennisetum hydridum	15.00	DM	27.13
Peanut straw	15.00	Metabolic energy, ME, MJ/kg	12.98
Corn	43.58	Crude protein, CP	14.82
Soybean meal	12.92	Ether extract, EE	3.97
Germ meal	8.61	Ash	11.57
Dicalcium phosphate	0.70	Neutral detergent fibre, NDF	44.12
Limestone	0.49	Acid detergent fibre, ADF	26.70
Sodium chloride	0.21	Calcium	0.90
Premix	3.50	Phosphorus	0.35
Total	100.00		

Premix provide followings per kg diet: Vitamin A 15000 IU, Vitamin D 5000 IU, Vitamin E 50 mg, Iron 90 mg, Copper 12.5 mg, zinc 100 mg, manganese 130 mg, selenium 0.3 mg, iodine 1.5 mg. All nutrients were measured except Metabolic energy.

### 2.2 Measurement of growth performance and feed efficiency

On the morning of the first day of the normal feeding period, all test goats were weighed on empty stomachs to determine their initial body weight. Subsequently, weight was measured every 30 days, and weight data were recorded. On the morning of the end of the experiment, the fasting weight was weighed as the final body weight to calculate the average daily gain. Feed and remaining feed of goats were recorded during the experiment, the average dry matter intake was calculated at the end of the experiment Dry matter intake was calculated by recording the daily dry matter intake of concentrate feed and roughage for 30 days and calculating the average value.

Morphometric measurements were taken at the beginning of the experimental period using a measuring tape and an adapted ruler. During the measurements, the lambs were maintained in an upright position on a concrete floor with no slopes. Zoometric indices were calculated from the relationships between morphometric measures. Thereafter, all goats were measured every 30 days including the index mentioned above. Body measurement traits are shown in Table 2. Body measurements included height, oblique length, chest girth, pipe circumference, waist angle width, and hip end width as described by Kusminanto et al. (12).

**Table 2:** Comparison of growth performance of Hainan Dong goat and Nubian goat after cross.

Items	Days	Dong goat	Nubian hybrid	SEM	*p*-value^a^
Initial body weight, kg	0	11.10	11.54	0.26	0.269
	30	13.23^a^	14.24^b^	0.33	0.044
	60	15.96	17.05	0.55	0.167
	90	18.59^a^	20.50^b^	0.50	<0.001
Average daily gain, g	0–30	95.13	80.19	8.97	0.315
	30–60	138.22	123.44	9.27	0.329
	60–90	93.51^a^	118.04^b^	4.44	<0.001
	0–90				
Dry matter intake, g	0–30	609.9^a^	728.27^b^	26.41	0.010
	30–60	883.25	931.06	25.69	0.236
	60–90	953.81^a^	1011.77^b^	13.84	<0.001
	0–90				
F/G	0–30	6.41^a^	9.08^b^	0.32	<0.001
	30–60	6.39^a^	7.54^b^	0.20	0.006
	60–90	10.20^a^	8.57^b^	0.13	<0.001
	0–90				
Body height, cm	0–30	6.58	7.24	0.45	0.353
	30–60	4.86	4.91	0.44	0.936
	60–90	4.38	3.95	0.38	0.486
Body oblique length, cm	0–30	5.24	5.97	0.70	0.488
	30–60	4.82	4.09	0.46	0.337
	60–90	5.07	4.88	0.41	0.779
Chest circumference, cm	0–30	2.86	2.77	0.45	0.892
	30–60	5.13	3.99	0.45	0.112
	60–90	3.51	3.44	0.39	0.905
Pipe circumference, cm	0–30	0.42	0.44	0.09	0.867
	30–60	0.45	0.34	0.06	0.275
	60–90	0.66	0.38	0.07	0.019
Waist Angle width (cm)	0–30	1.01	0.84	0.19	0.652
	30–60	1.37	1.08	0.19	0.392
	60–90	0.80	1.18	0.15	0.162
Hip end width (cm)	0–30	0.74	0.89	0.17	0.717
	30–60	1.59	1.77	0.21	0.582
	60–90	0.68	0.81	0.11	0.453

^a–b^ Values within a row with different superscripts differ significantly at p < 0.05 for items.

### 2.3 Slaughter and carcass characteristics

Slaughter was performed at the end of the test in a single batch (all mornings of the same day). The final weight of all animals was recorded after 12 h of fasting prior to slaughter (slaughter weight). Five goats were randomly selected from each group and 10 were slaughtered at the end of the 90 days period. After complete bleeding, the animals were sacrificed. The hot carcase weight was also recorded. The carcasses were split into two symmetrical parts using a saw. The meat quality was measured in the left half of each carcase. The surface area of a cross-section of *M. longissimus* dorsi (MLD) between the 12th and 13th ribs was obtained by tracing it onto acetate paper and measuring it using a planimeter. Slaughter rate was calculated as the ratio of slaughter weight to cold carcase weight. Then, the bones and meat of the carcase were separated, bone and meat weights were accurately weighed, and the meat-to-bone ratio, meat percentage, and carcase yield were calculated. Slaughter weight = live weight before slaughter; carcase weight refers to the weight of a slaughtered sheep that has been weighed after the removal of its fur, head, hooves, and entrails (retaining the kidneys and their surrounding fat); slaughter rate = carcase weight/slaughter weight×100%; meat-bone ratio = net meat weight/bone weight; meat percentage = net meat weight/slaughter weight × 100%; and carcase yield = net meat of carcase weight/carcass weight × 100%.

### 2.4 Colour, shear force, and cooking losses

Within 1 h after slaughter, meat colour parameters were recorded using the colorimeter (Minolta CR-410 Journal Pre-proof 8 colorimeter, Japan) technique, and scored as lightness (L*), redness (a*), and yellowness (b*) of the transverse section of longissimus dorsi after calibration with a standard white plate. The pH of the longissimus dorsi muscle was tested using a pH tester (German test 205) 45 min and 24 h after slaughter. Three parts of each sample were randomly selected for three measurements, and the average value was obtained. The muscle tissue was stored on ice at 4°C. The eye muscle area refers to the longissimus dorsi muscle at the 12th to 13th intercostal points of the carcase, which is cut crosswise. The cross-sectional outline of the eye muscle was drawn after the paper was covered with acid, and the eye muscle area was counted on a 1 mm^2^ standard calculation paper. Eye muscle area (cm^2^) = eye muscle height (cm) × eye muscle height (cm) × 0.7. Eye muscle pH means that the initial pH value of the carcase was measured with a direct carcase pH meter 45 min after slaughter, and the pH value of the carcase was measured for the second time after static acid discharge for 24 h, which was recorded as pH 2.4.

The longissimus dorsi muscle (2.5 × 2.5 × 7 cm) was removed, with its longest edge parallel to the direction of the muscle fibres. The muscle was put into a water bath at 80°C and boiled until the centre temperature of the muscle was 65°C. The meat was removed, cooled to room temperature, and sampled. During sampling, cylindrical meat pieces with a cross-sectional diameter of 1 cm were removed along the direction of the muscle fibres, and the shear force value was measured on a C-LM 3 B digital display muscle tenderness instrument. The final value was the mean of five readings per sample, expressed in Newtons (N). Shear force refers to removing the muscle membrane and fat tissue of Longissimus dorsi muscle and heating it in a water bath at 80°C, inserting a thermometer into the centre of the meat sample, taking it out when the centre temperature reaches 70°C, drying the surface moisture of the meat sample with filter paper, cooling it to room temperature, cutting 3 cm × 1 cm × 1 cm meat along the direction of muscle fibre development, and measuring it with a shear force tester. Repeat thrice for each meat sample was analysed in triplicate.

To calculate drip loss, three pieces of longissimus dorsi muscle with a size of 5 × 3 × 2 cm^3^were taken. Withing the longest edge parallel to the direction of muscle fibres, weighed, hung so that the direction of muscle fibres was perpendicular to the ground and putting in a inflated bag and stored in a refrigerator at 4°C for 24 h while avoiding the contact between the meat pieces and the bag. The drip loss was then calculated. After slaughter, 5–10 g longissimus dorsi muscle was collected within 1 h, accurate to 0.001 g, and placed in a constant temperature water bath at 85°C for 30 min. After removal, muscle surface moisture was drained and weighed. Cooked meat consumption rate was also calculated. Drip loss refers to accurately weighing the longissimus dorsi muscle of 3–4 g, cutting the meat into strips of 2 cm thick, 5 cm long and 3 cm wide, removing the outer muscle membrane, weighing the initial weight, recorded as G1, hanging in an inflatable plastic bag (muscle fibres down vertically) to ensure that the meat is not in contact with the plastic bag, and then hanging at 0°C to 4°C for 24 h. The filter paper was used to remove moisture from the surface of the meat sample and the weight was recorded as G2. The percentage of weight loss relative to the initial weight was calculated. Three replicates were analysed for each sample using the following formula:

Drip loss (%) = (G1 − G2) / G1 × 100%; Cooked meat rate refers to accurately weighing 5–10 g longissimus dorsi muscle, heating it in 85°C water bath for 40 min, taking it out, drying the surface moisture of the meat with filter paper, cooling it to room temperature and weighing it after cooking. Cooked meat percentage (%) = meat weight after cooking/meat weight before cooking × 100.

### 2.5 Muscle fibre characteristics

Images were acquired and analysed 3 traits of the muscle fibres, using an image analysis system (CaseViewer 2.4) to determine the three muscle fibre traits. Approximately 20 fibres without signs of tissue disruption were counted from a random field in each stained muscle sample section. Fibre diameter (μm), density (number/mm^2^) and fibre area percentages (μm^2^) were determined as previously described. Three 1 cm^3^ longissimus dorsi muscles were soaked and fixed in 4% paraformaldehyde solution, dehydrated, made transparent, impregnated with wax, embedded, sliced, stained, and scanned using a scanner (Pannoramic) for observation. Five 1 mm^2^ visual fields were randomly selected, and the number of muscle fibres in each visual field was determined to obtain muscle fibre density.

### 2.6 Rumen microbial analysis

The sample of 15 mL of ruminal fluid was filtered through 4 layers of cheesecloth and stored in −20°C until to microbiological analysis. Library construction and sequencing: After extracting total DNA from the samples, primers were designed according to the conserved regions. Sequencing adapters were added to the ends of the primers, the target sequences were amplified by PCR, the products were purified, quantified, and homogenised to obtain a sequencing library, and library QC was performed to construct libraries. Qualified libraries were sequenced using an Illumina Novaseq 6,000. The original image data files obtained by high-throughput sequencing (such as Illumina NovaSeq and other sequencing platforms) were converted into sequenced reads using base-calling analysis. The results were stored in the FASTQ (referred to as fq) format file, which contains sequence information of reads and their corresponding sequencing quality information (sequencing was carried out at Biomarker). The raw bioinformatics workflow data processing included the following three steps. First, raw reads filtration raw reads were filtered using Trimmomatic v0.33 Then the primer sequences were identified and removed using Cutadapt v1.9.1, which finally generated high-quality reads without primer sequences. Second, high-quality reads were assembled based on overlapping sequences using FLASH v1.2.7, which generated clean reads. Third, de-noise: De-noise was processed using dada2 (9) in QIIME2 2020.6 (10) to remove chimeric sequences, generating non-chimeric reads. Data analysis was performed using BMK Cloud.^1^

### 2.7 Data analysis

The *Student’s t-test* in SAS statistical software (SAS, Inst., Inc., Cary, NC, United States) was used to analyse the growth characteristics, carcase characteristics, and meat quality between the two groups. Statistical significance was set at *p* < 0.05. difference. Alpha diversity of the rumen microbial data between treatments was tested using the Kruskal-Wallis test and a *post hoc* Dunn Kruskal-Wallis multiple comparisons with a Bonferroni adjustment were used to evaluate differences between treatments with boxplotsbeing made in R (“ggpubr” packages). Beta diversity was visualised using a principal coordinate analysis plot. Featured bacteria among the treatments were identified using linear discriminant analysis (LDA) effect size (LEfSe).

## 3 Results

### 3.1 Growth performance

The growth performances of DG and NH under the same diet conditions during the fattening period are shown in Table 2. Body weight in the NH group was greater than that in the DG group at 30 and 90 days (*p* < 0.05). The average daily weight gain in the NH group was higher than that in the DG group during the 60–90 days (*p* < 0.05). Dry matter intake in the NH group was significantly higher than that in the DG group at–0–30 days and 60–90 days of age (*p* < 0.05). During 0–30 days and 30–60 days, the F/G ratio in the NHG group was higher than that in the DG group (*p* < 0.05), but lower than that in the DG group during the 60–90 days (*p* < 0.05). The pipe circumference of the NH group was lower than that of the DG group at 60–90 days of age (*p* < 0.05). No significant differences were detected in other growth performances between the two groups.

### 3.2 Slaughter performance

Slaughter weight was greater in the NH group than in the DG group (*p* < 0.05; Table 3). Similarly, the carcase weight was significantly higher in the NH group (*p* < 0.01). However, the meat-to-bone ratio and pH of the eye muscle in the NH group were significantly lower than those in the DG group (*p* < 0.05; *p* < 0.05). There were no significant differences in other indices of slaughter performance between the two groups. Other indicators of meat quality, such as muscle chroma (L), eye muscle area, and drip loss, were higher in the NH group than in the DG group; however, the difference was not statistically significant. The indices of muscle chroma (a), muscle chroma (b), eye muscle pH (24 h), shear force, and cooked meat rate in the NH group were lower than those in the DG group; however, no significant differences were found.

**Table 3:** Slaughter performance and meat quality.

Index’s		DG	NH	SEM	*p*-value^a^
Slaughter weight (kg)		18.59^a^	20.50^b^	0.62	0.036
Carcass weight (kg)		10.77^a^	12.25^b^	0.26	0.004
Slaughter rate (%)		55.16	54.37	1.01	0.599
Meat-bone ratio		2.54^a^	2.18^b^	0.09	0.025
Meat percentage (%)		34.77	33.34	0.65	0.154
Carcass yield (%)		63.06	61.33	0.67	0.108
Muscle chroma	a*	13.40	12.02	0.98	0.349
	b*	8.48	8.26	0.50	0.762
	L*	39.9	41.48	1.16	0.365
Eye muscle area (cm^2^)		11.46	14.41	0.49	0.214
Eye muscle pH		6.02^a^	5.64^b^	0.11	0.035
Eye muscle pH (24 h)		5.84	5.63	0.13	0.290
Shear force (*N*)		56.85	55.21	3.73	0.765
Drip loss (%)		1.21	1.89	0.37	0.229
Cooked meat rate (%)		72.59	69.57	1.19	0.111

^a–b^ Values within a row with different superscripts differ significantly at p < 0.05 for items.

### 3.3 Fibre morphology

**Figure 1:**
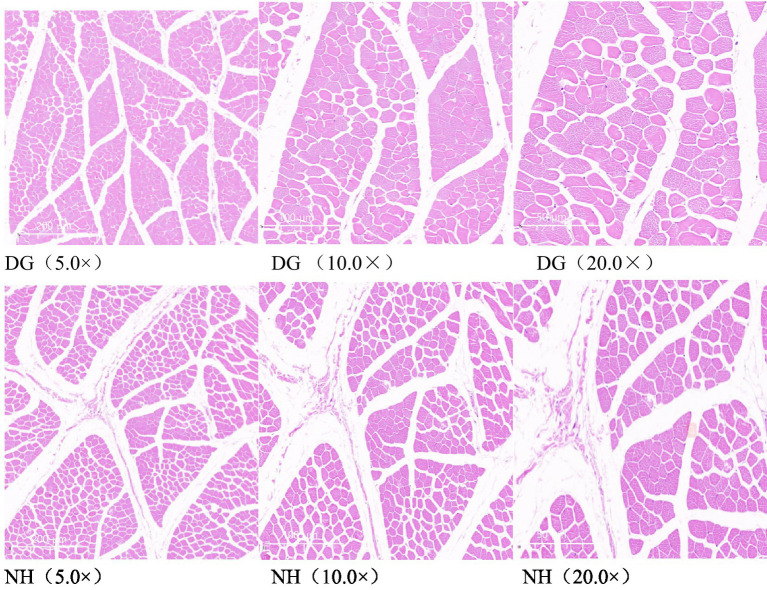
Hematoxylin and eosin staining. Scale bar = 200 μm, 100 μm, 50 μm, respectively.

To compare the morphology of the muscle fibres in the two groups, the representative characteristics of the two muscular tissues were investigated using different scale bars (Figure 1). The fibre diameter, fibre cross-sectional area, and fibre density of the muscles in the DG and NH groups are shown in Table 4. As shown in Table 4, the NH group had a smaller fibre cross-sectional area and fibre diameter than the DG group (*p* < 0.05). Fibre density was higher in the NH group than in the DG group (*p* < 0.0001).

**Table 4:** Comparison of muscle fibre morphology traits among two groups.

Muscle fibre morphology traits	DG	NH	SEM	*p*-value
Fibre density (number/mm^2^)	527.67^a^	706.87^b^	27.93	<0.001
Fibre cross-sectional area (μm^2^)	958.60^a^	662.61^b^	37.35	<0.001
Fibre diameter (μm)	117.31^a^	99.26^b^	2.48	<0.001

^a–b^ Values within a row with different superscripts differ significantly at p < 0.05 for items.

### 3.4 Rumen microbial communities

**Figure 2:**
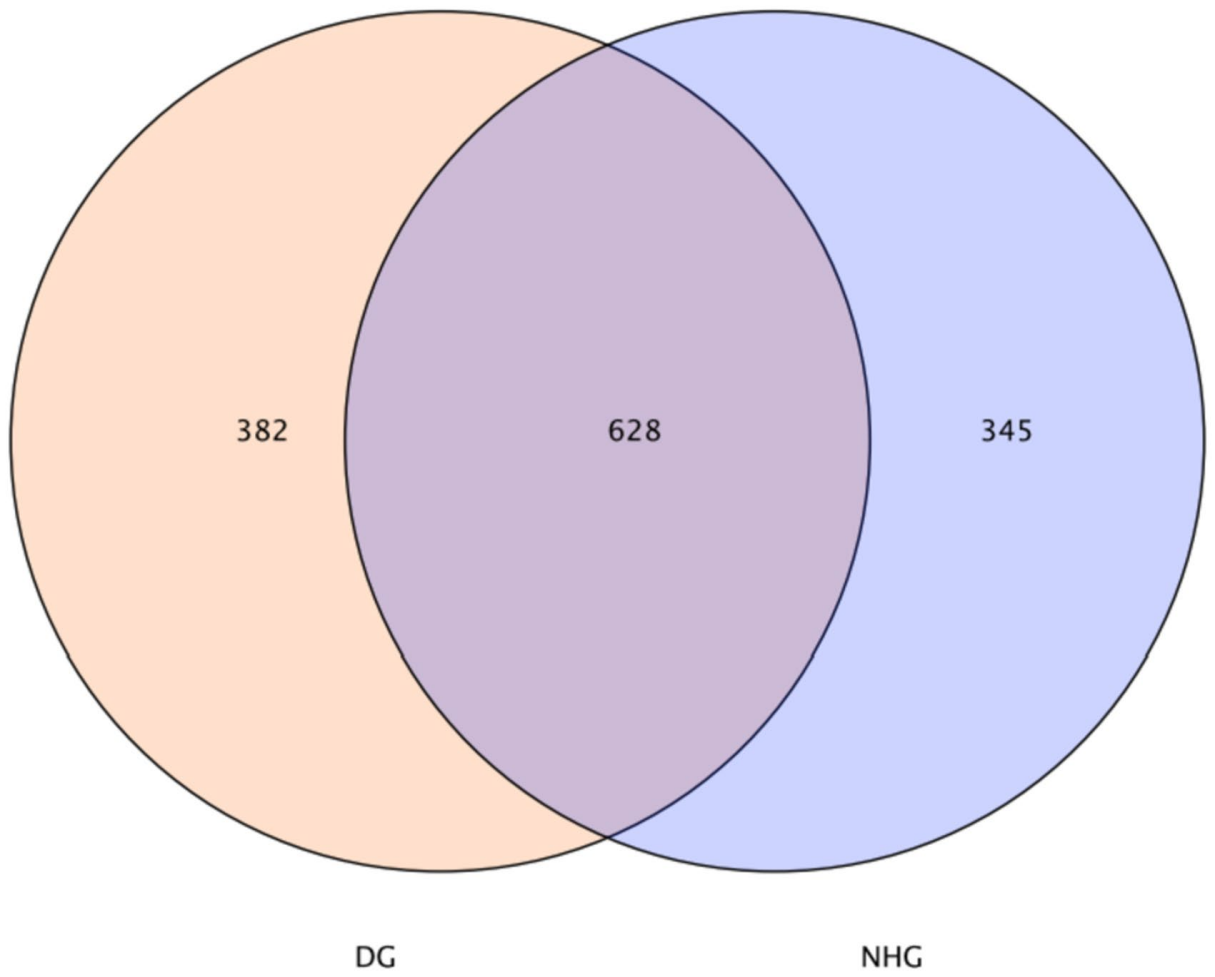
Venn diagram on features. DG, Dong goat; NHG, Nubian hybrid after cross.

In this study, common and unique features among samples (applicable to samples 2–5) were visualised using a Venn diagram, from which common microbes among groups could be identified depending on common features. The rumen had 628 OTUs, 382 unique OTUs in the DG group, and 345 unique OTUs in the hybrid group (NHG) (Figure 2).

**Figure 3:**
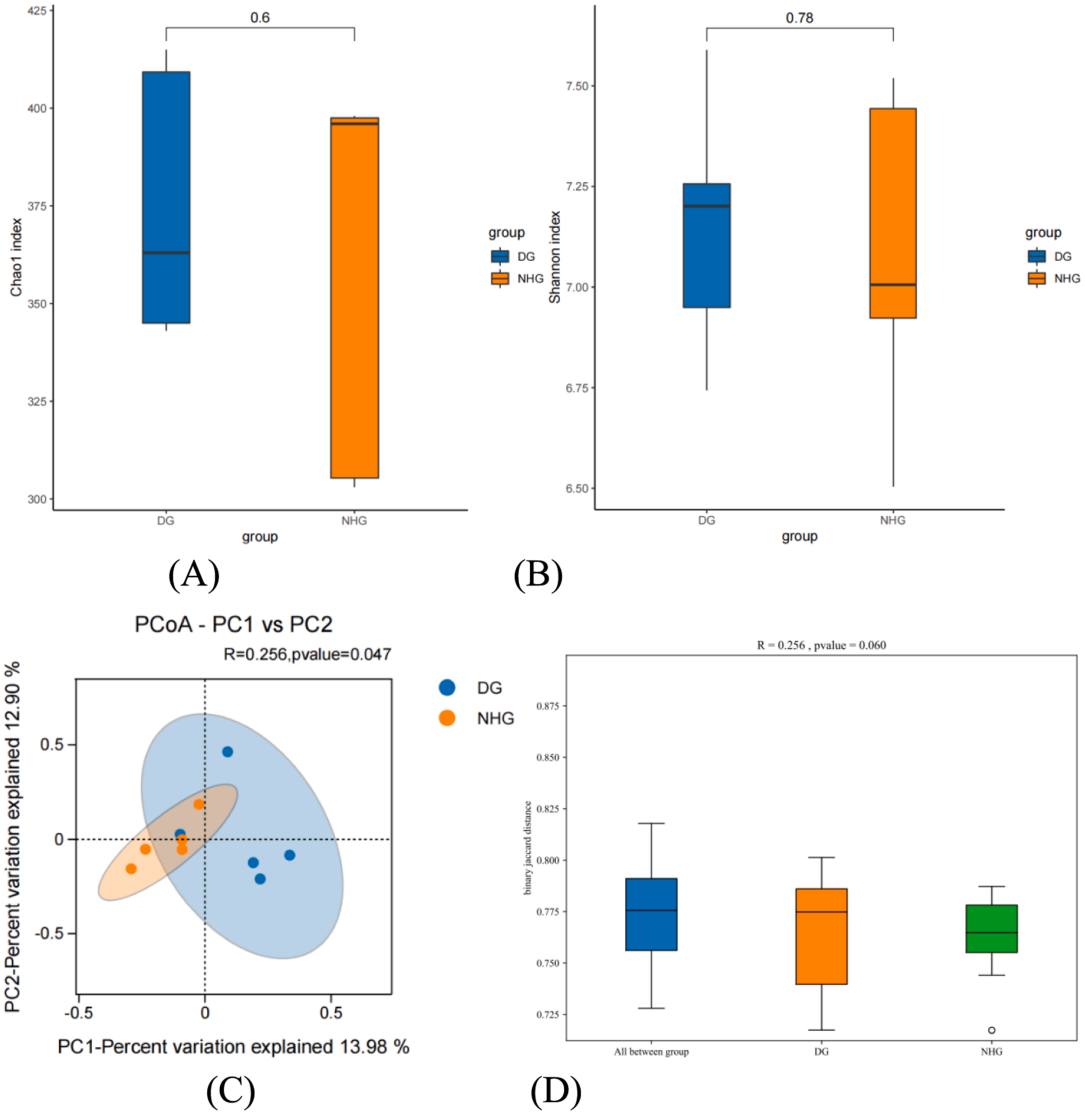
The different on microbial diversity and structure. DG, Dong goat; NHG, Nubian hybrid after cross. **(A)** The richness of rumen microbiota. **(B)** The diversity of rumen microbiota. **(C)** The principal coordinate analysis (PCoA) based on the bacterial binary_jaccard in each sample. **(D)** Analysis of Similarities (ANOSIM).

Alpha diversity reflects the species richness and diversity within a single sample. In the present study, there were no significant differences in the microbial richness (Chao1 index) or diversity (Shannon index) (Figures 3A,B) (*p* > 0.05). Beta diversity analysis was performed to compare the similarities between the different samples in terms of species diversity. Figure 3C shows the PCoA based on the system-generated tree non-weighted Unifrac algorithm. The abscissa of the figure is main component 1 (PC1), which contributes 13.98% to the representative difference in the total flora. The contribution rate of the ordinate principal component 2 (PC2) was 12.90%. The closer the samples were between the two groups, the greater was the similarity in composition. After adding the confidence ellipse, it can be seen that the control and hybrid groups overlap; that is, the two groups have similar flora and clustered similar flora. However, we did not observe distinct clusters of microbial communities among the groups according to the PCoA plot of beta diversity (*p* < 0.05) (Figure 3C).

ANOSIM (Analysis of Similarities, Figure 3D) was used to analyse whether beta diversity differed significantly between samples from different groups. An R value closer to 1 indicates that the between-group difference is greater than the within-group difference, a smaller R-value indicates no significant difference between and within groups, and a *p*-value less than 0.05 indicates high confidence in the test. A box plot of the test results is presented in Figure 3D. *p* > 0.05, R = 0.256 indicated that there were no significant differences between the hybridisation and control groups or between the two groups.

**Figure 4:**
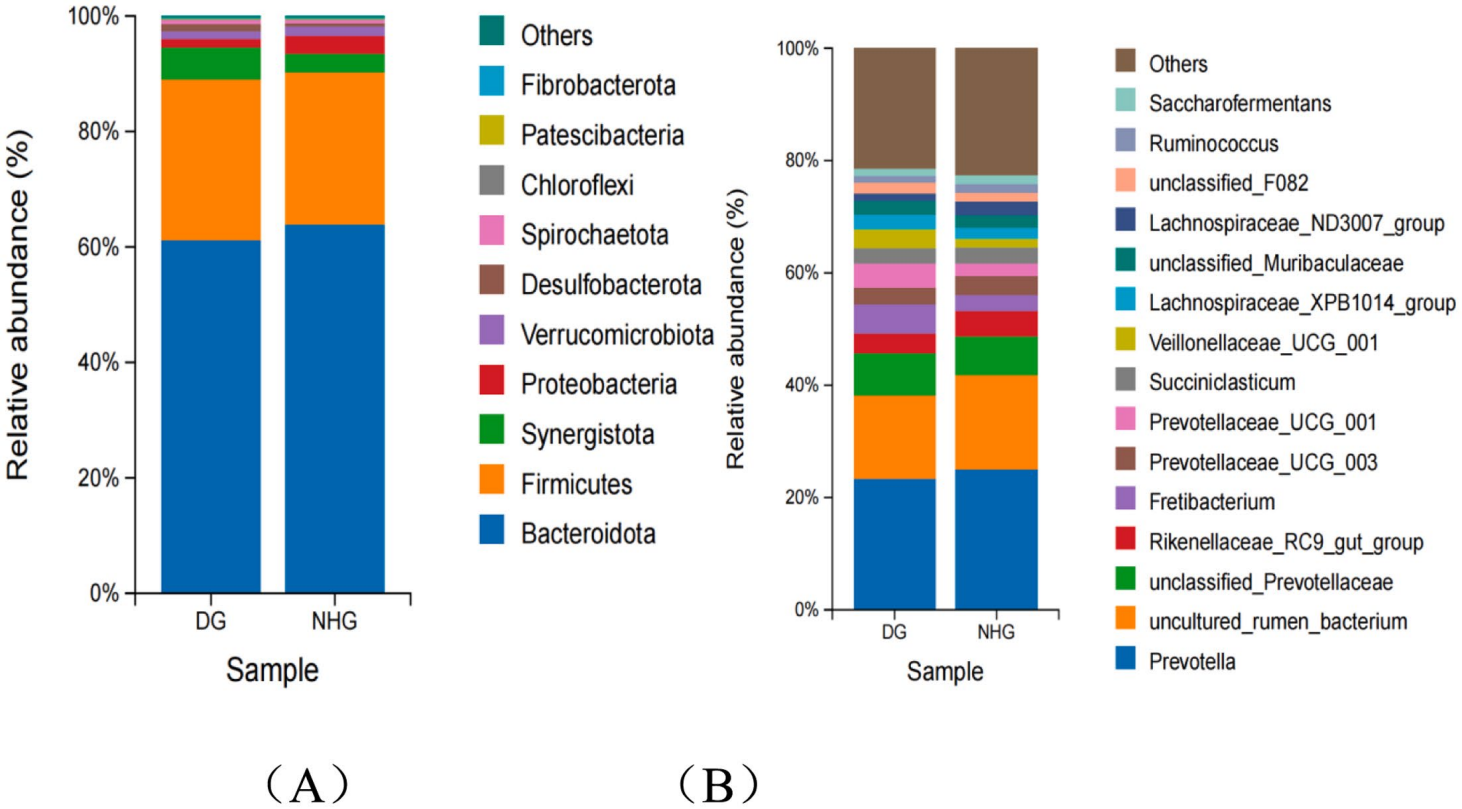
The composition of rumen microbiota at phylum level **(A)** and genus level **(B)**. DG, Dong goat; NHG, Nubian hybrid after cross.

In terms of rumen composition, 10 phyla had a relative abundance greater than 0.1% of the bacterial flora; at the phylum level, *Bacteroidetes* and *Firmicutes* were the dominant phyla between the two groups (Figure 4A). At the genus level, the top 15 bacterial genera in relative abundance were counted. *Prevotella*, *uncultured rumen bacterium*, *Rikenellaceae_RC9_gut_group*, the abundance of *unclassifed Prevotellaceae* and *Fretibacterium* were the top five genera. The relative abundance of *Prevotella, uncultured rumen bacterium and Rikenellaceae_RC9_gut_group* decreased (23.31% vs. 25.04%; 14.87% vs. 16.75%; 3.45% vs. 4.57%), while the abundance of *unclassifed Prevotellaceae* and *Fretibacterium* showed an opposite pattern (7.59% vs. 6.91%; 5.12% vs. 2.74%) (Figure 4B). As shown in Figure 4B, among the top 15 bacteria at the genus level, the first 10 genera accounted for 70.44 and 68.06% in the DG and NHG groups, respectively.

**Figure 5:**
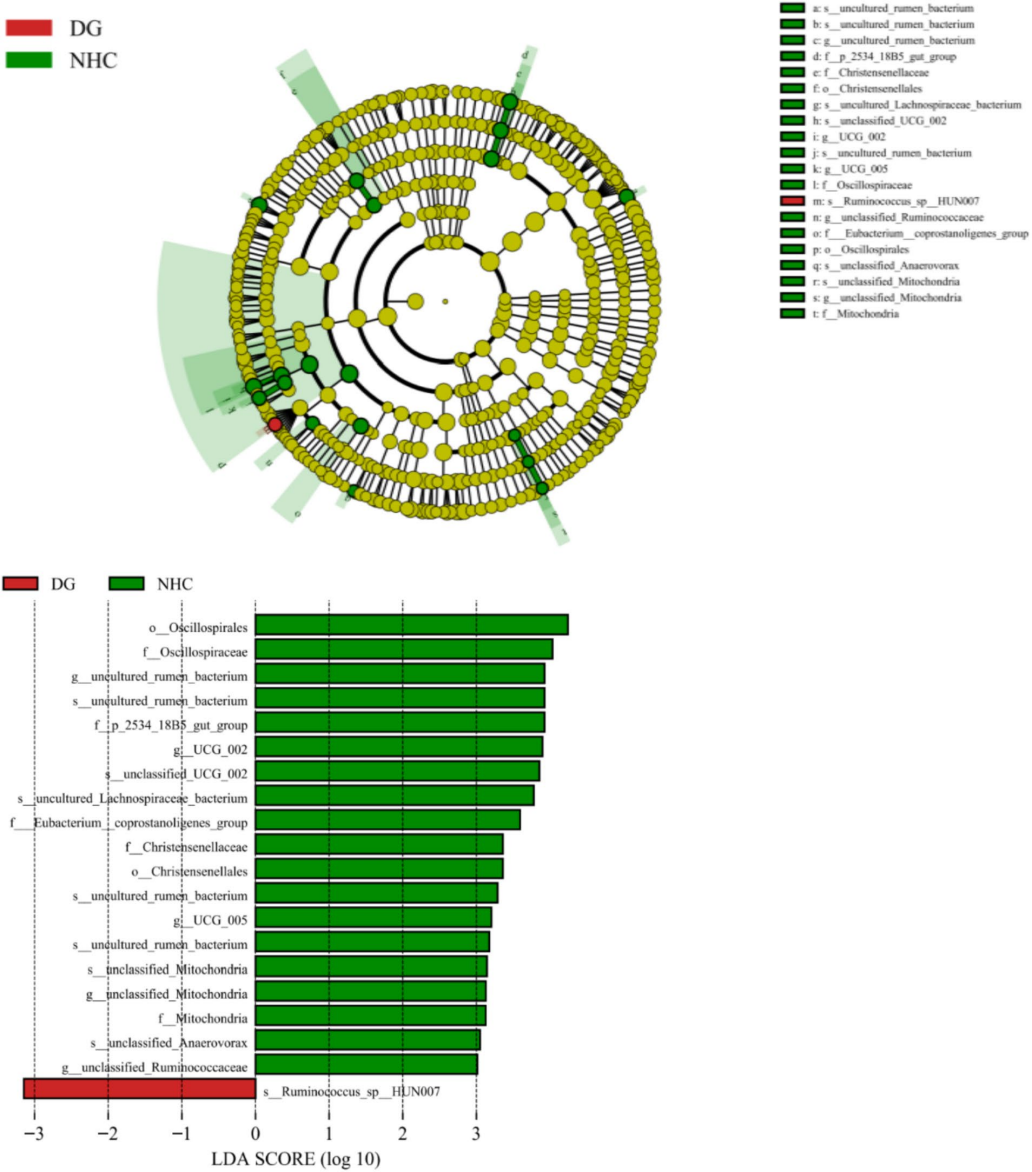
Cladogram based on LEfSe analysis. DG, Dong goat; NHG, Nubian hybrid after cross.

To identify the featured microbiota at the LDA = 4 level using LDA and LEfSe, the microbial composition differed between the two groups (Figure 5). In this way, we found biomarkers with statistical differences between the two groups and the evolutionary clade of the LDA distribution (Figure 5), which showed significant differences between groups in the microorganisms. The relative abundance of only one rumen species in the NHG group was significantly lower than that of the DG group, while the relative abundance of 2 orders, 5 family, 5 genus and 7 species was significantly higher than that of the DG group.

## 4 Discussion

In the present study, the differences in growth performance between two groups of goats under the same feeding environment and feed conditions were compared. The Hainan Dong goat, which is peculiar to Hainan province, is a local breed formed by long-term natural selection under distinct climatic conditions of high temperature and high humidity. It is well known not only for its rough feeding tolerance, strong disease resistance, and good adaptability to the tropical maritime climate in Hainan province (13), but also for its delicious meat with no smell of mutton, rich nutrition, and tenderness (5, 14). However, Hainan Dong goats have a slow growth rate and small size, resulting in a long feeding cycle, low carcase yield, and, ultimately, no appreciable economic benefit. Local goat farms are introducing larger, faster-growing exotic breeds to cross with local goats to increase economic returns to change that situation. Nubian goats are one of the most commonly used hybrids. Fortunately, according to the experimental results, the average daily gain of the experimental group (NHG) was indeed significantly higher than that of DG group, showing obvious growth rate advantage which is come ture that increasing the growth rate of native black goats by introduced the new breed. There is research found that breed type of kids affected average daily gain of kids at 30, 60 and 100 days significantly. Crossbred 1/2 Anglo-Nubian and 3/4 Anglo-Nubian kids showed growth rates in all investigated variables compared with pure Sahelian kids (15). This was probably due to the hybrid vigour of the crossing offspring. In our study, the advantage of the NHG group may have been positively correlated with the fact that the dry matter intake of goats in the NHG group was significantly higher than that of the DG group. At the same time, for the F/G ratio, the advantages and disadvantages of Dong goats are obvious; crossbreeding can effectively improve the efficiency of feeding and save costs with an increase in feeding days. The pipe circumference of the DG tended to be significantly higher than that of the NHG in the last month of the experiment, possibly because of the short stature of the native goats. In terms of production, the effect on slaughter performance and meat quality depends mainly on the feeding system and breed selection (16), particularly in the case of large-scale breeding. Significant differences (*p* < 0.05) in slaughter weight, carcase weight, and meat-to-bone ratio between the DG and NHG in slaughter performance were observed with the same feeding standard and breeding environment. There is research made A similar study reported that the pre-slaughter live weight, carcase weight, and meat-to-bone ratio of F1 hybrid black goats were better than those of pure Hainan black goats; the pre-slaughter live weight, carcase weight, meat weight, and bone weight of F1 hybrid black goats were 26.24, 31.79, 33.56, and 25.23% higher, respectively, than those of pure Hainan black goats (11). This suggests that the difference between the two at the genetic level is sufficient to manifest itself at the trait level. This also needs to be considered as another genetic source for the Nubian goat hybrid group. It is worth noting that although the weight of the NHG group was significantly greater than that of the DG group, the meat-to-bone ratio of the NHG group was significantly lower than that of the DG group, resulting in no significant differences in slaughter rate, meat percentage, or carcase yield. In connection with the feed intake of the two breeds, it may even be possible to infer the reasons why the local black goat breed was selected and favoured.

Muscle colour is mainly determined by myoglobin. Although the colour of meat is weakly correlated with its taste, the colour of meat in the market strongly influences consumer decisions (17). The DG group’s performance in muscle chroma brightness L*, redness a*, and yellowness b* was more in line with the evaluation of flesh colour than that of the NHG group (the higher the brightness, the better; the higher the redness, the better; and the lower the yellowness, the better). Huang et al. studied the hybrid improvement effect of Nubian and Longlin goats, and the results showed that the muscle colour of purebred Longbian and Nubian goats was not as good as that of the hybrid group; however, the three groups were at a normal level (18).

Although eye muscle pH at the time of slaughter was significantly different, the final pH difference disappeared 24 h after slaughter. This is related to glycogen degradation and lactic acid release before and after slaughter, and it can be reluctantly recognised that the process in the NHG group was slower than that in the DG group; however, in any case, the DG group was superior in both results. However, other studies have shown that the pH of muscles in the longissimus dorsi of Hainan black goats was lower than that of F1 hybrid black goats at 45 min or 24 h, indicating that the muscles of Hainan black goats are not easily preserved (11). The inconsistency between our results and those of Limin et al. may be due to the influence of meat storage during slaughter.

Shear force is an indicator of muscle tenderness: the higher the shear force, the less tender the lamb. Drip loss is an important parameter for measuring the water retention performance of muscles. Strong water retention performance, tender and jurious meat, dry and clean surfaces, poor water retention capacity, large water seepage on meat surface, and large amount of water loss during storage. The cooked meat rate is a measure of ripening loss; usually, meat with a high water content has a lower cooked meat rate. There were no statistically significant differences between the three data from the two groups, and subjective differences cannot be ruled out. The drip loss of the drip loss of F3 hybrid black goats was the highest. The cooked meat rate of Hainan black goats was higher in other studies, which is similar to our experimental results (11).

In this study, there were significant differences in the muscle fibre morphological traits of the longest dorsal muscle between the DG and NHG groups (*p* < 0.001). This is in line with the common sense that different breeds have different traits, and understanding the differences in genotypes is important for optimising the performance and meat quality of different sheep breeds (19). It has been reported that a larger fibre cross-sectional area or diameter results in higher carcase weight and fat content (20). The results of the present study are inconsistent with their conclusions. The greater the muscle fibre density, the lower the fat content, and the fibre density of the DG group was significantly lower than that of the NHG group; that is, the fat content of the DG group was higher. The fibre cross-sectional area and diameter were consistent with our observations.

The fibre cross-sectional area and fibre diameter of the dorsal longus muscle of the DG group were significantly larger than those of the NHG group. However, the carcase weight of the DG group was significantly lower than that of the NHC group. Considering that the meat-to-bone ratio of the DG group was significantly greater than that of the NHG group, and that the slaughter and carcase yields of the DG group were slightly higher than those of the NHG group, we believe that the muscle fibre morphology traits are more reflected in the proportion than in the numerical size.

In this study, the DG group was a Hainan native Dong goat, whereas the NHG group was a Nubian goat and Hainan Dong goat cross under the same feeding standards and breeding environment. The analysis of the two groups of rumen microorganisms was based on species taxonomy, phylum and genus level analysis, alpha diversity analysis, and beta diversity analysis, reflecting the differences between the two groups of unique or common microorganisms, species richness and diversity, and similarity in species diversity. This was used to compare ruminal microorganisms in the same feeding environment in different goat breeds. This may have significance for production practices in large-scale farming.

General studies suggest that the differences produced by ruminant species in their rumen microorganisms are not absolute; for example, in experimental animals grazed on the same natural pasture, there were clear, distinct differences in the composition of anaerobic rumen fungi between yak and these two sheep species, whereas no such separation was found between Tibetan sheep and Small Tail Han sheep (21). On the other hand, considering the feeding environment, for example, in the study by Holstein and Hanwall, genetic factors interacted with the feeding system, leading to divergent effects in the rumen and, therefore, to large differences in microbial gene abundance (22). This study also leans towards the views mentioned above.

At the phylum level, the phyla with a relative abundance of microflora greater than 0.1% were consistent, and the two groups had Bacteroidetes as the dominant phylum. Similarly, at the genus level, the proportions of the first 10 genera in the two groups were similar, with Prevotella being the first dominant genus in the two groups. In the alpha diversity analysis, there was no significant difference (*p* > 0.05) between the two groups in terms of species abundance or diversity. In the beta diversity analysis, it was also proved that the two groups of flora were similar and the flora clustered similarly.

Although there is a degree of mutual selection between microbes and hosts, this was not revealed in this study, and the underlying explanation for this may be that they cannot satisfy the host physiology of the shared microbiota between the two largest animals as a contributing factor. The correlation between feed sources and rumen flora has been demonstrated, and the results of this study show that the same diet has similar effects on rumen microorganisms, which in turn have related effects on growth and production performance (23). However, it is also possible that, in this experiment, the sample size of microorganisms sent for testing was not representative, resulting in insignificant differences. The specific reasons for this need to be verified further.

## 5 Conclusion

Overall, Hainan Dong goats showed significant improvements in dry matter intake, average daily gain, slaughter weight, and carcase weight after crossing with Nubian goats. However, the meat-to-bone ratio is not advantageous. The crossbreeding technique had a great effect on the muscle fibres of Hainan Dong goats, whereas it had little effect on the composition of rumen microflora. The current study revealed that crossbreeding of Nubian goats with Dong goats has the potential to be used in a wide range of applications owing to its effectiveness in increasing production efficiency.

## Data Availability

The datasets presented in this study can be found in online repositories. The names of the repository/repositories and accession number(s) can be found in the article/supplementary material.

## Footnotes

^1^ www.biocloud.net

## References

[1] Álvarez C Koolman L Whelan M Moloney A. Effect of pre-slaughter Practises and early post-mortem interventions on sheep meat tenderness and its impact on microbial status. Foods. (2022) 11:181. doi: 10.3390/foods11020181 35053913 PMC8775201

[2] Xu TS Zhang XH Gu LH Zhou HL Rong G Sun WP. Identification and characterization of genes related to the development of skeletal muscle in the Hainan black goat. Biosci Biotechnol Biochem. (2012) 76:238–44. doi: 10.1271/bbb.110461, PMID: 22313753

[3] Miao Q Huang SY Qin SY Yu X Yang Y Yang JF. Genetic characterization of toxoplasma Gondii in Yunnan black goats (*Capra Hircus*) in Southwest China by Pcr-Rflp. Parasit Vectors. (2015) 8:57. doi: 10.1186/s13071-015-0673-0, PMID: 25622613 PMC4316756

[4] Zhao Y Zhang J Zhao E Zhang X Liu X Zhang N. Mitochondrial DNA diversity and origins of domestic goats in Southwest China (excluding Tibet). Small Rumin Res. (2011) 95:40–7. doi: 10.1016/j.smallrumres.2010.09.004

[5] Wang D Zhou L Zhou H Hou G Li M Shi L. Effects of nutrition level of concentrate-based diets on growth performance and carcass characteristics of Hainan black goats. Trop Anim Health Prod. (2014) 46:783–8. Epub 2014/03/04. doi: 10.1007/s11250-014-0565-x, PMID: 24585343

[6] Wilson RT. Small ruminant production and the small ruminant genetic resource in tropical Africa. Rome: Food and Agriculture Organization of the United Nations. (1991).

[7] Stemmer A Siegmund-Schultze M Gall C Zárate A. Development and worldwide distribution of the Anglo Nubian goat. Trop Subtrop Agroecosystems. (2009) 11:185–8.

[8] Yuan C Guo T-T Liu J-B Yaojing Y Yang B-H. Conservation and utilization of indigenous goats and breeding of new breeds in China. In Simões J Gutiérrez C editors. Sustainable Goat Production in Adverse Environments. Vol. 1. Springer Cham (2017). p. 457–472.

[9] Meza-Herrera CA Menendez-Buxadera A Serradilla JM Lopez-Villalobos N Baena-Manzano F. Estimates of genetic parameters and Heterosis for birth weight, one-month weight and litter size at birth in five goat breeds. Small Rumin Res. (2019) 174:19–25. doi: 10.1016/j.smallrumres.2019.02.018

[10] Zhang S Zhang Q Yang L Gao X Chen T Li T. Comparative and functional analysis of Mirnas and Mrnas involved in muscle Fiber hypertrophy of juvenile and adult goats. Genes. (2023) 14:315. doi: 10.3390/genes14020315 36833242 PMC9956283

[11] Limin W Ruiping S Yan L Ruina L Quanwei L Xinli Z. Slaughtering performance and meat quality of Hainan black goat and its hybrid Offsprings with Nubian black goat. Anim Husb Feed Sci. (2019) 11:49–52. doi: 10.19578/j.cnki.ahfs.2019.02.002

[12] Kusminanto RY Alawiansyah A Pramono A Sutarno CM. Body weight and body measurement characteristics of seven goat breeds in Indonesia. IOP Conf Ser Earth Environ Sci. (2020) 478:012039. doi: 10.1088/1755-1315/478/1/012039

[13] Wang D Zhou L Zhou H Hou G Shi L. Effects of dietary Α-lipoic acid on carcass characteristics, antioxidant capability and meat quality in Hainan black goats. Ital J Anim Sci. (2017) 16:61–7. doi: 10.1080/1828051X.2016.1263546

[14] Hu J Zhao W Niu L Wang L Li L Zhang H. Gene Organization and characterization of the complete mitochondrial genome of Hainan black goat (*Capra Hircus*). Mitochondrial DNA A DNA Mapp Seq Anal. (2016) 27:1656–7. doi: 10.3109/19401736.2014.958715, PMID: 25211090

[15] Momani MS Sanogo S Coulibaly D Al-Olofi S Alkhewani T. Growth performance and Milk yield in Sahelian × Anglo-Nubian goats following crossbreeding in the semi-arid zone of Mali. Agricultura Tropica et Subtropica. (2012) 45:117–25. doi: 10.2478/v10295-012-0020-9

[16] Ates S Keles G Demirci U Dogan S Kirbas M Filley SJ. The effects of feeding system and breed on the performance and meat quality of weaned lambs. Small Rumin Res. (2020) 192:106225. doi: 10.1016/j.smallrumres.2020.106225

[17] Zhang C Luo J Yu B Zheng P Huang Z Mao X. Dietary resveratrol supplementation improves meat quality of finishing pigs through changing muscle Fiber characteristics and Antioxidative status. Meat Sci. (2015) 102:15–21. doi: 10.1016/j.meatsci.2014.11.014, PMID: 25524822

[18] Huang HZJ Li Y Lu J Huang H Zhu Z. Hybrid improvement effect of Nubian goat and Longlin goat. Exp Res. (2022) 2:1–4. doi: 10.3969/j.issn.2096-3637.2022.02.001

[19] Cheng S Wang X Zhang Q He Y Zhang X Yang L. Comparative transcriptome analysis identifying the different molecular genetic markers related to production performance and meat quality in longissimus Dorsi tissues of mg × Sth and Sth sheep. Genes. (2020) 11:183. doi: 10.3390/genes11020183, PMID: 32050672 PMC7074365

[20] Shen J Zhen H Li L Zhang Y Wang J Hu J. Identification and characterization of circular Rnas in longissimus Dorsi muscle tissue from two goat breeds using Rna-Seq. Mol Genet Genomics. (2022) 297:817–31. doi: 10.1007/s00438-022-01887-1, PMID: 35429278

[21] Guo W Wang W Bi S Long R Ullah F Shafiq M. Characterization of anaerobic rumen fungal community composition in yak, Tibetan sheep and small tail Han sheep grazing on the Qinghai-Tibetan plateau. Animals. (2020) 10:144. doi: 10.3390/ani10010144, PMID: 31963125 PMC7023293

[22] Bharanidharan R Lee CH Thirugnanasambantham K Ibidhi R Woo YW Lee HG. Feeding systems and host breeds influence ruminal fermentation, methane production, microbial diversity and metagenomic gene abundance. Front Microbiol. (2021) 12:701081. doi: 10.3389/fmicb.2021.701081, PMID: 34354694 PMC8329423

[23] Costa-Roura S Balcells J de la Fuente G Mora-Gil J Llanes N Villalba D. Nutrient utilization efficiency, ruminal fermentation and microbial Community in Holstein Bulls fed Concentrate-Based Diets with different forage source. Anim Feed Sci Technol. (2020) 269:114662. doi: 10.1016/j.anifeedsci.2020.114662

